# An Overview of Systematic Reviews to Inform the Institutional Design of Scientific Advisory Committees

**DOI:** 10.1002/gch2.201800019

**Published:** 2018-08-08

**Authors:** Asha Behdinan, Elliot Gunn, Prativa Baral, Lathika Sritharan, Patrick Fafard, Steven J. Hoffman

**Affiliations:** ^1^ Global Strategy Lab York University/University of Ottawa Canada; ^2^ Faculty of Medicine University of Toronto Toronto Ontario Canada; ^3^ Dahdaleh Institute for Global Health Research Faculty of Health and Osgoode Hall Law School York University Toronto Ontario Canada; ^4^ Graduate School of Public and International Affairs University of Ottawa Ottawa Ontario Canada

**Keywords:** advisory boards, effectiveness of scientific advisory committees, institutional designs, scientific advisory committees, systematic review

## Abstract

The current lack of synthesized evidence for informing the design of scientific advisory committees (SACs) is surprising in light of the widespread use of SACs throughout decision‐making processes. While existing research points to the importance of quality, relevance, and legitimacy for SACs' effectiveness, those planning SACs would benefit from efforts to systematically pinpoint optimal designs of these committees for maximal effectiveness. Search strategies are developed for seven electronic databases. Of the 1895 systematic reviews identified, six reviews meet the inclusion criteria: they report the results of systematic reviews that followed a clearly identified systematic methodology, examine factors related to the design of SACs, and involve processes in the natural or social sciences. These reviews collectively summarize 444 primary studies. Three of the six reviews look at the impacts of SAC size, two evaluate the influence of the committee's diversity, and half mention the importance of properly on‐boarding new members. The goal is to identify recurring themes to understand the specific institutional features that optimize the usefulness of SACs. In turn, this overview of systematic reviews aims to contribute to a growing body of literature on how SACs should be designed to maximize their effectiveness and helpfulness for decision‐making.

## 1 Introduction

As suggested in the introductory paper of this series, a scientific advisory committee (SAC) is defined as a group of individuals with relevant expertise that provides decision‐makers with advice predominantly based on research evidence from either the natural or social sciences. SACs have many different names and forms, such as technical committees, expert advisory groups, science boards, and, if conceived broadly, can sometimes include ethics review boards, citizen panels, parliamentary committees, royal commissions and public inquiries.^1^, ^2^ However, despite the widespread use of SACs for providing policymakers and practitioners with advice to inform their decisions, there is currently a lack of synthesized evidence that can inform the design of these bodies to maximize their effectiveness.^3^ An effective SAC is one that “increases the chances that those who have power to make binding decisions […] have the opportunity to consider all of the relevant evidence.”^4^

While seemingly narrow in scope, SACs are used in a vast number of processes, sectors and contexts.^5^, ^6^ Many authorities commission advice from SACs, including governments, multilateral organizations, and private businesses. SACs can be evaluated based on the effectiveness of their advice, particularly with regards to how helpful they may be for informing key decisions. There is some research on the determinants of SAC effectiveness, which points to the importance of design features that ensure advice is viewed as being high‐quality, relevant and legitimate.^2^, ^7^, ^8^ These design features include size, diversity, credibility, transparency, and decision‐making procedures, among many others.^7^, ^9^, ^10^

Despite the literature currently available on this topic, there has been little to no emphasis placed on the design of SACs as a tool to optimize effectiveness.^6^ And yet, many challenges appear in the current designs of SACs as they stand: being trapped in groupthink processes, collective shirking, lack of leadership, and conflicts of interest and transparency.^11^ By identifying, appraising and synthesizing existing systematic reviews relevant to the institutional design of SACs, this overview distils lessons from a broad swath of research literature that can help convenors of SACs to ensure these groups have a greater chance of being effective. Ultimately, this overview of systematic reviews identifies and aims to inform which institutional features of SACs maximize their effectiveness.

## 2 Results

**Figure 1:**
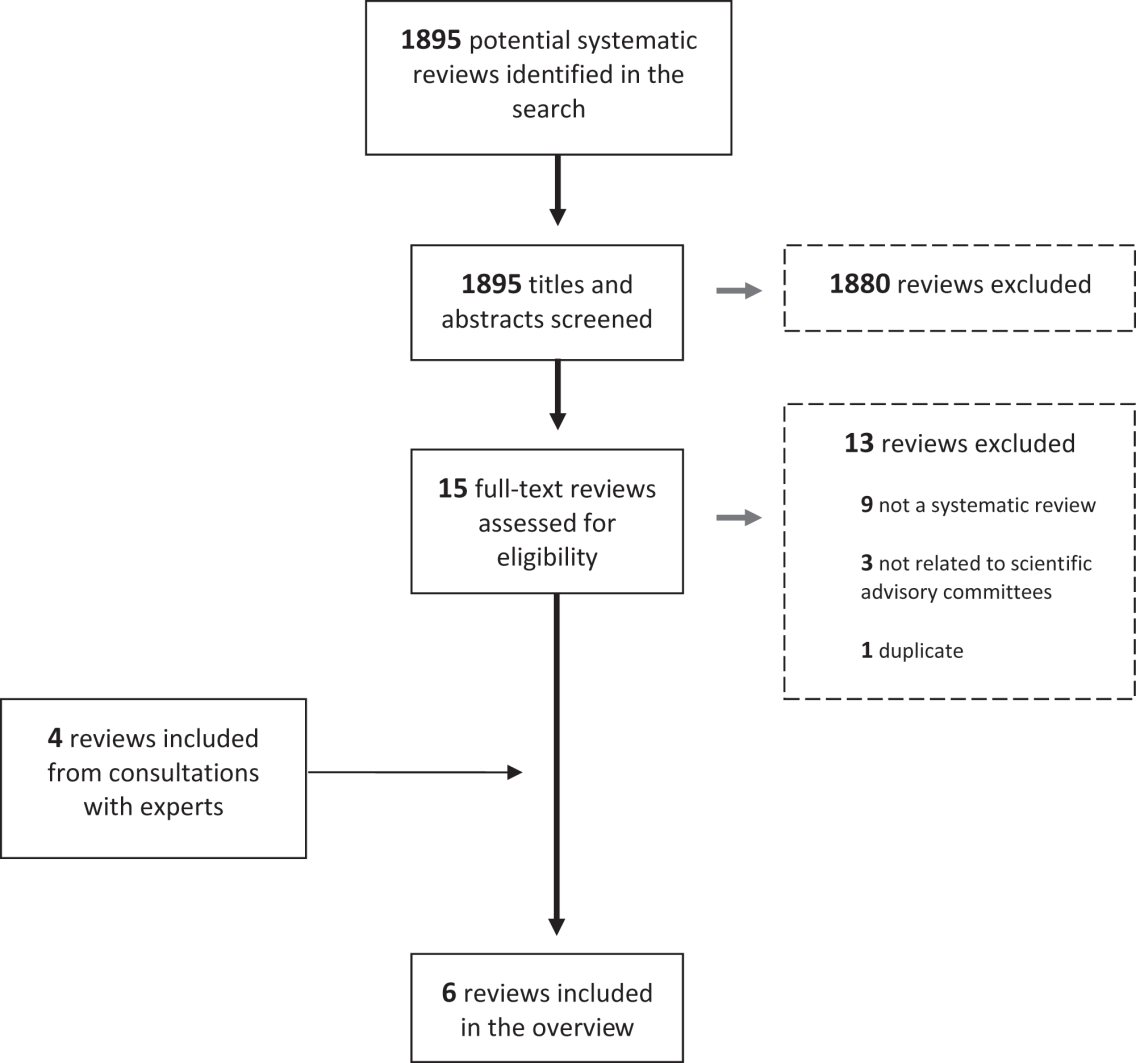
PRISMA flow diagram showing the process of review selection.

A total of 1895 potential systematic reviews were identified through the search of seven electronic databases. Two research team members screened the titles and abstracts and yielded 15 reviews that met the inclusion criteria. The search was repeated after one month and no new results were found. Additionally, four reviews were identified by experts in the field. Full‐text screening of the 19 reviews yielded a total of six systematic reviews, as outlined in **Figure** **1**. Of the 19 reviews identified, reviews were excluded during the full‐text screening process either because of duplication (*n* = 1), not following a systematic methodology (*n* = 9), or not meeting the criteria of a “scientific advisory committee” (*n* = 3). All the reviews identified had AMSTAR ratings on the lower end of the spectrum: the overall quality of the six reviews was evenly split between low (score 0–4) and moderate (score 5–8) (**Table** **1**).

**Table 1:** Studies identified at each stage of the review

Source	Number of abstracts assessed	Number of relevant abstracts	Number of full papers assessed	Number of papers included in the review
OVID	782*	12	12	1
Sociological Abstracts	130	0	0	0
SCOPUS	983	3	3	1
Subtotal	1895	15	15	2
Other sources	0	0	4	4
Total	1895	15	19	6

^*^ The number of abstracts assessed for the individual OVID databases include: EMBASE: 98, PsychINFO: 5, Cochrane: 7, Joanna Briggs: 2 and MEDLINE: 670, for a total of 782.

Four of the six reviews addressed only optimal design features for SACs, one solely addressed SAC impact on decision‐making bodies, and one analyzed both. Two of these reviews focused on SACs establishing clinical practice guidelines, two on consensus development methods, two on citizen and patient panels, and one on small group processes in data monitoring committees. The reviews are summarized in **Table** **2**.

**Table 2:** Full text screening results (included reviews only)

Systematic review	Studies included	Focus	Population	Review type	AMSTAR ratinga)	Summary of findings	Outcomes
Hutchings and Raine (2006)	52 (1996–2004)	Consensus development methods	Usage in health care	Systematic review	2/11	Identifies how key factors affect the recommendations produced by formal consensus development methods. Multispecialty groups are more likely to consider a wider range of opinions. There is little evidence to generalize how the characteristics of groups affect recommendations produced.	Identified four different factors that impact decision‐making: regional differences, international differences, specialty mix, and different methods.
Murphy et al. (1998)	177 (1966–1996)	Consensus development methods	Usage in clinical guideline creation	Narrative	1/11	Identifies the factors that influence decisions from three consensus development methods (Delphi method, nominal group technique, and consensus development conference). Cue selection should be made explicit. Participant background should be reflective of target population to increase credibility. There is no consensus on the best method for synthesizing judgments.	Identified the five most essential parts of consensus development: questions, participants, information, method, and output.
Nilsen et al. (2013)	6 (1806–2009)	Methods of consumer involvement	Healthcare consumers (patients, community organizations)	Systematic review; RCTs only	6/11	Identifies how to best involve consumers in healthcare decisions at the population level by looking at RCTs. Consumer involvement can improve relevance of patient information material, and a face‐to‐face meeting is more engaging which in turn affects community health priorities. Consumer input does not impact understanding of informed consent documents.	Assessed quality of outcomes in material produced, knowledge attained, and survey results.
Légaré et al. (2011)	71 (Beginning–2009)	Clinical practice guidelines	Patient and public involvement programs	Systematic review	5/11	Identifies how patient and public involvement programs (PPIPs) are used to develop and implement clinical practice guidelines (CPGs). PPIPs are most often used to integrate patients' values in CPG guidelines. They principally put forward recommendations and revise drafts.	Analyzed studies by factors that presented barriers or facilitators to creating PPIG in implementing CPGs.
Walker et al. (2004)	57 (1950–2002)	Small group processes relevant to data monitoring committees	Data monitoring committees in the lab or real‐world settings	Systematic review	5/11	Identifies factors behind erroneous decisions reached by data monitoring committees (DMCs). Biased leadership or presentation of information, limited range of opinions expressed, and poor procedures for handling information increase error rates. DMCs should be diverse, led by experienced and impartial chairs, and follow a predefined analysis plan.	Identified the ten factors that increase likelihood of DMCs making wrong decisions
Bertens et al. (2013)	81 (Beginning–2012)	Panel (expert, consensus) diagnosis	Diagnostic studies	Systematic review	3/11	Identifies methods used in panel diagnoses. Most studies were unclear about critical aspects of panel diagnosis; guidelines were issued for future reporting involving panel diagnosis.	Identified methods of panel diagnosis and areas for improvement.

^a)^ The following four reviews were reviewed by a third reviewer for AMSTAR ratings: Hutchings and Raine (2006), Murphy et al. (1998), Légaré et al. (2011), and Bertens et al. (2013).

### 2.1 Optimal Design Features for SACs

The four reviews that examined characteristics of SACs highlighted many key features of successful committees. Hutchings and Raine^12^ focused primarily on the rules in decision‐making that underline methods in formal consensus development. They structured their review of 52 studies around three widely‐used methods: the nominal group technique (NGT), the Delphi survey, and the RAND/UCLA appropriateness method. Murphy et al.^13^ structured their review of 177 studies around the identification of the five most essential features of consensus development to establish clinical guidelines: questions, participants, information, method, and output. Légaré et al.^14^ discovered that only a limited number of studies dealt with the essential features, resources, and impact of patient and public involvement programs (PPIPs) on clinical practice guidelines. In addition, they allege that no study examined health outcomes at all. The 71 studies tallied by Légaré et al. focused more on qualitative assessments of participants' experiences. Finally, Walker et al.^15^ reviewed 57 articles in the social science literature regarding small group processes that are involved in decision‐making. They identified factors influencing outcomes for decision‐making bodies, and concluded with implications for data monitoring committees (DMCs), i.e., experts who lead and regularly assess clinical trials.

The four reviews pointed to several core themes that determine the quality, relevance, and legitimacy of SACs. The most prominent key design factors influencing the impact of SACs include consensus development methods, the number of individuals in the committee, the diversity of individuals within the committees, training and oversight, and finally, the methods of communication implemented.

Consensus development methods are unique in providing a multiround setting in order to produce and revise scientific opinions as novel information is introduced to the literature. First, individual results are aggregated to produce an overall group verdict. Second, a structured feedback interaction takes place, where individuals are given an opportunity to change their initial choices. Consensus building methods was found to be an important feature in two studies. Lower consensus was found to reflect division over controversial issues, such as the appropriateness of a novel medical treatment (Hutchings et al.). This variation may also be an indication to the differences in categorization employed by studies to create rankings or ratings (dichotomous vs ordered). Interestingly, this variation stayed consistent regardless of the comparative groups' designs. It was also found that cues embedded in the questions used for consensus development can shape both individual and group‐level clinical judgments. For example, explicit cues may crowd out other important features, and as a result, participants may not be aware of the importance they place on specific cues.

Several studies pointed to the size of the committee as being an important feature in determining the effectiveness of SACs. According to Murphey et al., groups with fewer than six individuals showed less reliability, but diminishing returns were seen in groups consisting of twelve or more participants. Légaré et al. also suggested this, albeit from the perspective of patient and public involvement programs, stating that small size necessarily constrains their ability to be representative of the population at large. Walker et al., also list size as one of the ten factors associated with poor decision‐making quality, such as “groupthink,” i.e., the tendency to conform to the majority's opinion within a group. In short, across these studies, it is evident that groups under 12 members had a lower potential for such adverse decision‐making qualities, but a group must be sufficiently large to ensure optimal credibility.

Along with group size, the diversity in committee composition was also discussed by two of the included reviews. Overall, multispecialty groups performed better than their single‐specialty counterparts, because participants are able to learn from one another and moderate differences accordingly. The authors concluded that methodological variation led to inconsistent findings in the direction of bias in groups from different countries. That is, there is “little generalizable evidence” for how formal consensus development methods influence, and are in turn influenced by, varying types of participating groups and individuals. In addition, homogenous groups consisting of participants with the same specialty background leaned toward extreme choices rather than an overall general agreement post‐discussion. In short, studies indicated that heterogeneity is an optimal design feature to prevent bias in recommendations and for ease of group moderation.

Group members' training and insight into the topic, as well as the structure, procedures, and oversight of the committee itself were also noted to be of importance in the design of SACs, as indicated by two of the included studies. Murphey et al. pointed to informational influences having a relatively greater impact than normative influences in fact‐oriented groups. One particular study in Murphy et al. discovered that the introduction of novel information had the highest probability in changing the opinions of individuals and groups. Légaré et al. also suggested several pertinent takeaways: training can boost participation confidence when faced with complex and technical medical evidence and/or terminology; other helpful items referred by the authors include a well‐trained knowledgeable staff, descriptive documents, and clear expectations regarding roles and responsibilities. Ultimately, the introduction of new information without prior training or familiarity could pose barriers to effective decision‐making, which may be ameliorated by detailed procedural and technical documents and clearly‐defined member roles.

Finally, the importance of clear communication within committees was alluded to in all of the included reviews. Appropriate communication minimizes both procedural and technical knowledge gaps between the diverse group members. Conversely, the inhibitive effect of poor communication such as social loafing, unequal participation, negative framing of information and an unstructured format are all associated with poor decision‐making quality.^14^

### 2.2 SAC Effectiveness

The overall effectiveness of a SAC would appear to be a function of 1) the way the input of the group members is aggregated; 2), the inclusion of inputs from the public; and 3), asking several individuals to independently assess the same body of evidence.

A critical determining factor for the overall effectiveness of a SAC is the process for aggregating the input of the group, both individually and collectively. In Murphy et al.'s review of 177 studies,^2^ they state that the aggregation of group output is determined in two stages: 1) the weight given to the contributions of individual participants, 2) the calculation of the group's degree of agreement, based on the contributions, regardless of weight. Murphy et al. specified that voting, the most popular approach to achieving consensus, is preferable in situations that require ranking between options; voting is not suggested for normative judgments. The authors state that voting itself has been found to be paradoxical at times (e.g., emergence of alliances), to the detriment of the larger outcomes. They also suggest that the evidence is inconclusive on the benefits of weighting by expertise, and that the definitions of group agreement do not appear to impact the actual level of agreement.

Another factor influencing the effectiveness of a SAC is whether or not the expertise of the group is supplemented by input from the public. Nilsen et al.^16^ included six randomized control trials (RCTs) consisting of a total of 2123 participants. They concluded that while RCTs can be useful (e.g., “feasible”) for obtaining evidence on the importance of consumer consultation in healthcare decisions, the six RCTs reviewed have unfortunately been scored with a moderate or high degree of bias. As such, conclusions drawn are impacted by these methodological limitations. One included study by Chumbley et al. pointed to consumer consultation via medical leaflets. This led to improved patient satisfaction with only a small associated increase in anxiety, suggesting that the inclusion of consumers in the development process may improve the quality and accessibility of a product. This is consistent with a large qualitative literature that argues for public input into the work of expert panels.^17^, ^18^

Finally, we found one systematic review that made the case for asking several individuals to assess the same body of evidence with a view to controlling for bias. Bertens et al.^19^ examined 81 articles on panel diagnosis, also known as “consensus diagnosis” and “expert panel diagnosis.” This is an increasingly popular clinical practice whereby the clinician utilizes multiple test results to reach a final diagnosis. No known preferred method of conducting a panel diagnosis currently exists. As such, the authors performed a systematic review on reported cases to describe trends, assess quality, and issue recommendations. Bertens et al. reported that larger panel sizes may ultimately help prevent inaccuracy in final diagnoses. This is consistent with the other studies that emphasized the merit of large, multispecialty SACs.

## 3 Discussion

### 3.1 Key Themes

The synthesis of evidence collected highlight several key themes, namely moderate group size, multidisciplinary group composition, established group protocols, and adequate training and communication that determine the quality, relevance and legitimacy of SAC effectiveness.

One of the overarching themes is the matter of committee size and the balancing act required: too many members in a committee lead to a deleterious impact with members conforming to the majority whereas too few members lead to a non‐representative cohort of the population. While no specific committee size was consistently reported by the selected systematic reviews, it is recommended that SACs be composed of six to twelve members to ensure that both representation and the communication of unique perspectives are achieved.

Another important feature in the design of SACs is to ensure the presence of member diversity. It is important that SACs reflect different specialties^2^, ^12^ as well as diversity in demographic characteristics, expertise, and initial views on the subject matter, in order to optimize the performance of SACs. This heterogeneity will likely ensure that the committee does not lean toward a biased consensus or a single extreme, and that members are able to learn from different perspectives in order to achieve a holistic conclusion. However, it should be noted that with the exception of participant specialty background, there is a lack of generalizable evidence^12^ regarding how group features influence recommendations proposed by a SAC.

Another key area of interest includes the presence of key procedural determinants of SAC structure. In order to achieve effective decision‐making processes, it is recommended that SAC structure is maintained through the presence of clarifying protocols which delineate clear responsibilities, a structured group format, and a clear framework of the task at hand.

Finally, communication between the SACs and its staff, particularly with regards to training and facilitating cohesion between committee members is vital to ensure optimal operation of SACs. It is also recommended that training and support in the form of appropriate email and phone correspondence, a supportive chair, as well as clearly outlined objectives and group processes be offered to SAC members. In specific citizen panel contexts, mentoring and open lines of assistance are also key in optimizing SAC performance.

In addition to the main themes, the following specific insights were observed: Group dynamics influence consensus development. Multispeciality groups facilitate learning, even in the absence of face‐to‐face interaction.^20^ Yet single‐specialty groups such as consumer consultation can increase the quality of decision‐making.^16^ Group decision‐making is moderated by processes such as alliances and exchange of information, and is negatively affected by members who are overly insulated from external sources of information.^2^ Groups composed of experts from multiple fields did not perform as optimally when working on technically‐demanding material. In highly technical contexts, variation in levels of expertise among participants can present a barrier to optimal performance. Training of SAC members can help bridge knowledge gaps among different participants and increase self‐awareness of participant‐driven bias, while ensuring that the group remain representative of the population‐at‐large.^14^, ^15^ Experienced leaders and facilitators are encouraged to make use of pre‐specified analysis plans to mitigate errors and resolve any conflicts that arise.^21^, ^22^

This overview also reveals significant gaps in the literature, as evident by the dearth of evidence for SAC features in non‐health fields, and the paucity of studies focusing on other design features, such as credibility, transparency, and specific decision‐making protocols. Wilsdon and others have pointed to the need for research on the internal workings of science advice.^23^, ^27^ While there is a great deal of recent work on science advice and the role of evidence in public policy more generally,^28^ much of it is country‐specific, little of it isolates the particular challenges associated with SACs, and there are few systematic reviews. Thus, the research reported here confirms that there is a significant need for research to be conducted on specific SAC features. In particular, additional research is required to understand the relationship between the impact of SAC design (and its effectiveness) and relevant variables of interest such as policy, practice and populations as well as governance and monitoring. Moreover, while there is ample research in health sciences – five out of the six included reviews focused on this field – there were very few studies identified that discussed SACs beyond the health sector. Furthermore, there is clear evidence of the sheer lack of high quality systematic reviews: this broad search strategy was able to identify only six out of 1895, highlighting the need for more studies to review design features of SACs. Finally, another key gap identified is the lack of research on how these design features and processes affect the eventual implementation of the recommendations made by these groups. Only two out of the six systematic reviews^16^, ^21^ discussed the impact of SACs on decision‐making bodies. That is, more evidence is needed to draw sound conclusions on the effectiveness of SACs.

Ultimately, while many key insights were gained through the analysis of the reviews, the small number of reviews identified has limited our ability to form strong, specific, evidence‐based recommendations. Nonetheless, several key insights have been drawn that can have a positive impact on the functioning and impact of SACs. We recommend that SACs include a minimum of six and a maximum of twelve members. Committees need to be large enough to encourage discussion and representation, but not too large as to lead to collective shirking. Communication was also noted to be a significant factor in SAC success. Thus we recommend that training and support be provided for committee members, as well as clearly delineated protocols and procedures for the group. In the same vein, the consequences of heterogeneity within SACs may pose a barrier to the group achieving their optimal performance. In order to overcome this barrier, we suggest implementing training measures and appointing experienced facilitators to fill gaps in knowledge and procedure, as suggested by the gathered evidence. However, conflicting data from the reviews prevents us from forming a concrete recommendation with regards to the optimal diversity within a SAC.

### 3.2 Strengths and Limitations of the Study

There are several key strengths in the design and methodology of this overview. First, we conducted an extensive literature search to address the research question of SAC effectiveness. Second, the search strategy employed (see Appendix Appendix 1) was sufficiently broad enough to yield a high number of potential reviews—nearly 1900 studies. In addition, due to the variety of databases consulted, a high level of diversity within the review results was observed. Third, the overview was undertaken methodologically with a high level of transparency, whereby results were analyzed by two independent research team members. These measures ensured that independent biases were limited. Finally, each review that met the delineated inclusion criteria underwent quality assessment in order to ensure a high level of confidence in the conclusions drawn. Ultimately, since this overview does not exclude reviews based on context or discipline, recommendations that can be applied broadly to SACs are made. The overarching goal of the paper is to serve as a starting point for further research investigation on how these committees can best be optimized, based on their specific goals and purposes.

One of the main limitations of this study is the number of reviews identified. While the results of the search led to the discovery of a key gap in the SACs research literature, a greater number of available reviews would have led to a more comprehensive overview with clearer recommendations. In addition, many of the reviews identified had AMSTAR ratings on the lower end of the spectrum, reducing a level of confidence of the conclusions drawn. This overview also excluded studies that may have shed light on key factors for group decision‐making outside of the SAC context. The search was also limited to reviews published only in English. In addition, by focusing only on systematic reviews, the scope for this overview remains quite broad. This, however, represents the trade‐off between identifying broad‐spectrum recommendations versus context‐specific advice; more targeted investigations would yield conclusions that better fit the unique environments in which specific groups operate in. Finally, we chose to keep SACs designed for both policy and practice, even if the respective dynamics are different in each case. In other words, given the limited number of reviewed identified, we chose to keep the SACs for clinical practice guidelines.

### 3.3 Future Research Directions

While this type of study should allow for the drawing of broad lessons on SAC features, the small number of systematic reviews that met the inclusion criteria limits its explanatory power. A different study design that included a broader range of studies (e.g., narrative reviews; case studies; etc.) might generate a broader and richer set of recommendations, albeit based on a weaker evidence base. There is a rich diversity of studies of scientific advisory committees and science advice more generally.^24^, ^25^, ^26^, ^27^ Moreover, there is a growing literature on the role of scientific evidence in policy making^28^, ^29^, ^30^ as well as a body of theory and empirical studies on policy advisory systems both of which could prove useful.^31^, ^32^ A careful analysis of this research could yield additional insights into the optimal design and operation of SACs.

## 4 Conclusion

The results and recommendations of this study should help inform some key decisions in the way SACs can be designed to improve their effectiveness. This is important given the widespread use of SACs across so many processes, sectors and contexts. This overview also highlights key gaps in current knowledge about SACs, identified promising areas for future inquiry, and calls for additional systematic reviews of relevant evidence upon which future overviews could build. With further research and synthesis, clearer recommendations for how to institutionally design SACS to optimize their effectiveness should be possible. In the meantime, there are at least 444 primary studies and six systematic reviews from which convenors of SACs can learn.

## 5 Experimental Section

An overview of systematic reviews was conducted to synthesize evidence from across a range of contexts. Systematic reviews collate relevant empirical evidence from individual studies using explicit, systematic methods to answer a specific research question.^33^ The main advantages of systematic reviews are that they can overcome important limitations inherent in traditional or narrative summaries of research. Because they impose discipline on the review process, they can uncover associations not previously identified, maximize transparency, and thereby minimize bias.^34^ In turn, overviews of systematic reviews compile information from multiple systematic reviews relevant to a single topic using the same systematic methodology.^33^ Whereas a single systematic review tends to be more narrowly focused, an overview of systematic reviews can synthesize a much broader swath of research evidence to explore the bigger picture, including commenting and making recommendations on what works across settings and designs. Conducting a systematic review of reviews ensures that these recommendations are rooted in patterns and evidence seen to be significant as synthesized by the review methodology. By transparently searching, assessing and summarizing the available evidence, the lessons regarding currently known topics can be offered and the gaps in the areas of further research can be identified.

*Search Strategy*: A generic search strategy was developed in consultation with one health science librarian and one social science librarian from the University of Toronto to identify systematic reviews that were relevant to the institutional design of SACs (see **Appendix** **1**). The strategy to identify these reviews included search terms related to:

- SACs (including synonymous labels such as expert, technical, panel, and body);
- Design features, processes and outputs (including group procedures, consensus, and decision‐making); and
- Systematic reviews and meta‐analyses.

Since this overview was conducted with no a priori hypotheses on whether particular institutional designs were effective, search terms related to effectiveness were not included in order to maximize sensitivity (i.e., identify the largest number of relevant reviews possible). But overall, SAC effectiveness refers to how certain features can affect SAC decision‐making, whether their recommendations are adopted, and how they impact the stakeholders involved.

The generic search strategy was adapted for the following electronic databases: Medline (1946–present), EMBASE (1947–present), and Cochrane Database (1992–present) for biomedical literature, PsycINFO (1887–present) for psychology literature, Sociological Abstracts (1952–present) for social science literature, SCOPUS (1995–present) for interdisciplinary science literature, and Joanna Briggs (1999–present) for multidisciplinary systematic reviews. Searches were executed on February 28, 2017. Experts in the field were consulted to identify additional systematic reviews that were not found in the search, or to include reviews that had not yet be published. The tailored search strategies for each database can be found in Appendix Appendix 1.

*Selection of Systematic Reviews*: Two members of the research team (AB/EG) independently screened the title and abstract of each identified record for inclusion. Systematic reviews were selected during the title/abstract screening if the designated research team members reached consensus that the following inclusion three criteria were met:

1. Included reviews must follow a clearly identified systematic methodology.
2. Included reviews must examine and draw conclusions and key lessons on the effectiveness of one or more design features of SACs.
3. Included reviews must examine SACs design within a natural science or social science context.

Reviews that focused solely on teamwork, team‐building, and group decision‐making, as well as those that were not published in English were excluded. Reviews were not excluded based on their dates of publication. Reviews that did not employ systematic methodology were excluded, given that the primary aim of the study was to broadly distill common themes on the features which strengthen SACs. Systematic reviews were solely included in the overview to ensure that the existing evidence may be applied broadly to any SACs, as opposed to non‐systematic reviews that may pertain to specific fields only.

Disagreements on review inclusion were resolved through discussion between the two research team members (AB/EG). In case a consensus could not be reached, a third team member was consulted (LS). The full text of those reviews that met the inclusion criteria during the title/abstract screening were assessed by the same two research team members, using the same inclusion and exclusion criteria mentioned above. Figure 1 shows the number of records that were included and excluded at each stage of screening.

*Data Extraction*: Following full‐text screening, qualitative data were extracted from each included review, including the type of SAC discussed, the field in which the SAC was operating, and the SAC's main objectives. The main findings from each review, including their strengths and limitations, were independently extracted, discussed and then reconciled and combined by the two designated research team members. A standardized form, found in Table 2, delineates the research team's focus when extracting data from each review. These include: the number and publication date of included primary studies in the systematic review; the research question and context of each review; the target population; the analytic approach used in the systematic review; and finally, the main results pertaining to the key factors of interest described below.

*Data Analysis and Synthesis*: The findings from the reviews were organized across studies and analyzed under two overarching themes. The primary focus of analysis focused on institutional design features and proximal determinants of SAC efficacy. These outcomes include a) institutional design features, such as diversity, size, transparency, and decision‐making procedures; and b) proximal determinants of SAC effectiveness, namely quality, relevance and legitimacy. This overview will also secondarily examine the impact of the SACs themselves, and the effectiveness of recommendations they made. Particularly, the review analysis involved: a) any beneficial or adverse outcomes related to the impact of SACs; and b) measures of its effectiveness in terms of impact of decisions made by the SAC's commissioning body. In other words, the reviews were assessed to determine the impact of SAC recommendations and, in turn, the effectiveness of these recommendations once implemented. Effectiveness was observed through two means: 1) the adoption and the implementation of the recommendations by the SAC's commissioning body; or 2) the lack of unintended consequences as a result of the recommendations. Results from the analysis of each individual review were then compared and assessed for recurring patterns to determine optimal design features for SACs that can be either generalizable or context‐specific.

*Assessment of Quality*: The included reviews were assessed for their quality using AMSTAR, a tool which evaluates systematic reviews according to 11 criteria. A high‐quality review is defined as having met 9–11 criteria, a medium‐quality review will have met 5–8, and a low‐quality review will have met 0–4. Two research team members completed quality assessment independently; a third independent reviewer (LS) was consulted when the two initial ratings were incongruent.^35^

## Appendix 1

### Generic Search Strategy

**Table:** 

Theme	Search terms
Scientific advisory committees	1. Scientific advisory committee.tw 2. ((Scientific or expert or technical) adj3 (committee or body or panel or board or commission or group*)).tw. 3. Advisory committees 4. ((Committee or body or panel or board or commission or group) adj (methods or construction or creation or design or develop* or effective*)).tw.
Institutional design features	5. Consensus 6. Group processes 7. Group structure 8. Decision making 9. Consultation 10. Transparency 11. Diversity 12. Size
Outputs	13. Delivery of health care 14. Guideline
SAC and design features	15. (1 or 2 or 3 or 4) and (5 or 6 or 7 or 8 or 9 or 10 or 11 or 12)
SAC and outputs	16. (1 or 2 or 3 or 4) and (13 or 14)
	17. 15 or 16
Meta‐analysis or systematic review filter	18. limit 17 to (Meta‐analysis or systematic reviews)

### Appendix 2

Examples of database‐specific search strategies.

### Sociological Abstract

ALL((scientific advisory committee) OR ((scientific or expert or technical) adj (committee or body or panel or board or commission or group*)) OR (advisory committees) OR ((committee or body or panel or board or commission or group) adj (methods or construction or creation or design or develop* or effective*))) AND ALL(consensus or group processes or group structure or decision making or consultation or transparency or diversity or size) OR ALL((scientific advisory committee) OR ((scientific or expert or technical) adj (committee or body or panel or board or commission or group*)) OR (advisory committees) OR ((committee or body or panel or board or commission or group) adj (methods or construction or creation or design or develop* or effective*))) AND ALL((delivery of health care) or (guideline)) AND ALL((meta‐analysis) or (systematic review)).

### Scopus

(Title‐Abs‐Key((“scientific advisory committee”) OR ((scientific OR expert OR technical) W/0 (committee OR body OR panel OR board OR commission OR group*)) OR (“advisory committees”) OR ((committee OR body OR panel OR board OR commission OR group) W/0 (methods OR construction OR creation OR design OR develop* OR effective*))) AND Title‐Abs‐Key(consensus OR group AND processes OR group AND structure OR decision AND making OR consultation OR transparency OR diversity OR size)) OR (Title‐Abs‐Key((“scientific advisory committee”) OR ((scientific OR expert OR technical) W/0 (committee OR body OR panel OR board OR commission OR group*)) OR (“advisory committees”) OR ((committee OR body OR panel OR board OR commission OR group) W/0 (methods OR construction OR creation OR design OR develop* OR effective*))) AND Title‐Abs‐Key((“delivery of health care”) OR (guideline)))AND Title‐Abs‐Key((“meta‐analysis”) OR (“systematic review”)).

## Conflict of Interest

The authors declare no conflict of interest.
